# Cyclophilin D deficiency exacerbates diet-induced obesity and selectively enhances skeletal muscle mitochondrial respiration in male mice

**DOI:** 10.1007/s00424-026-03218-4

**Published:** 2026-10-13

**Authors:** Jazzmin L. Hembree, William A. Abplanalp, John W. Elrod, Paul T. Pfluger

**Affiliations:** 1 Department of Microbiology, Miami University, Oxford, OH USA; 2 The Heart Institute, Division of Cardiothoracic Surgery, Cincinnati Children’s Hospital Medical Center, The University of Cincinnati College of Medicine, Cincinnati, OH USA; 3 Aging + Cardiovascular Discovery Center, Department of Cardiovascular Sciences, Lewis Katz School of Medicine at Temple University, Philadelphia, PA USA; 4 Research Unit NeuroBiology of Diabetes, Helmholtz Munich, Neuherberg, Germany; 5 Division of NeuroBiology of Diabetes, TUM School of Medicine & Health, Technical University of Munich, Munich, Germany; 6 German Center for Diabetes Research (DZD), Neuherberg, Germany

## Abstract

Mitochondrial dysfunction is a hallmark of obesity-associated metabolic disease, with the mitochondrial permeability transition pore serving as a key regulator of cellular energy homeostasis. Cyclophilin D, encoded by *Ppif*, is the principal regulatory component of this pore and has been implicated in insulin secretion, adipocyte survival, and whole-body energy balance. However, whether *Ppif* loss produces a consistent metabolic phenotype when challenged with high fat diet (HFD) exposure, and whether systemic metabolic alterations reflect tissue-specific changes in mitochondrial oxidative capacity, remains unresolved. Here, we characterized male, 21-weeks-old mice with germline *Ppif* deletion longitudinally over 34 weeks under standard (SD) and HFD using body composition analysis, indirect calorimetry, glucose and insulin tolerance testing and tissue-resolved, Seahorse-based mitochondrial respirometry. Under SD, knockout mice displayed elevated fat mass without differences in food intake or energy expenditure. High-fat feeding progressively amplified this adiposity, with knockout mice developing significantly greater body weight and fat mass than wild-type littermates by weeks 28 and 36. Glucose tolerance and insulin sensitivity remained comparable between genotypes under either diet. ANCOVA revealed significant effects of lean mass (*p* < 0.001) and genotype (*p* = 0.017) on energy expenditure, as well as a significant lean mass × genotype interaction (*p* = 0.025). Consistent with a distinct contribution of lean mass to EE in KO vs. WT mice, we found increased ADP-stimulated mitochondrial respiration in quadriceps of knockout mice under both diets, without corresponding differences in brown adipose tissue or liver, or changes in mitochondrial content across tissues. These findings establish cyclophilin D as a diet- and tissue-specific regulator of energy homeostasis, informing therapeutic strategies targeting the permeability transition pore or cyclophilin D interactome in metabolic disease.

## Introduction

Obesity and its associated metabolic complications, including type 2 diabetes, dyslipidemia, and cardiovascular disease, represent a growing global health burden that is driven in large part by dysregulated energy homeostasis and adipose tissue expansion. At the cellular level, mitochondria act as key regulators of energy metabolism, and mitochondrial dysfunction is strongly implicated in the development of diet‑induced obesity and insulin resistance [1, 2]. A key regulator of mitochondrial integrity is the mitochondrial permeability transition pore (mPTP), a high-conductance channel whose aberrant or sustained opening leads to dissipation of the mitochondrial membrane potential, release of pro-apoptotic factors, and disruption of oxidative phosphorylation [3]. Understanding the molecular mechanisms that govern mPTP activity and their downstream metabolic consequences has therefore become an important area of investigation in the context of metabolic disease.

Cyclophilin D (CypD), encoded by the *Ppif* gene, is a matrix peptidyl-prolyl cis-trans isomerase that serves as a critical facilitator of mPTP opening by directly binding to pore constituents including the adenine nucleotide translocator and components for oxidative stress-induced opening, thereby acting as key modulator of mPTP sensitivity and mitochondrial stress responses [4, 5]. Beyond its canonical role in cell death regulation, CypD has emerged as a broader regulator of mitochondrial metabolism, with implications for whole-body energy balance and glucose homeostasis [6, 7]. However, reports on its metabolic role are highly divergent, with studies describing protection from, susceptibility to, or altered progression of diet‑induced obesity and glucose intolerance in CypD‑deficient mice. These discrepancies highlight the need to clarify how loss of CypD influences whole‑body metabolic regulation. Furthermore, whether alterations in whole-body metabolic parameters in CypD‑deficient mice are mechanistically linked to tissue-specific changes in mitochondrial oxidative capacity has not been fully elucidated.

Here, we characterize the metabolic phenotype of male whole-body *Ppif* knockout mice undergoing SD and HFD exposure for 34 weeks, incorporating body composition analysis, comprehensive indirect calorimetry, glucose and insulin tolerance testing, and mitochondrial respiratory function measurements across metabolically distinct tissues. Our findings demonstrate that CypD deficiency exacerbates diet-induced obesity in a dietary context-dependent manner, selectively affecting adiposity, energy expenditure, and skeletal muscle mitochondrial respiration, thereby establishing a tissue- and diet-specific role for CypD in the regulation of whole-body metabolic homeostasis.

## Methods

### Mouse experiments

Generation of *Ppif* knockout mice has been described previously [4]. *Ppif* knockout mice were maintained on a mixed C57BL/6:129 genetic background, with unknown Nnt status (Jackson Lab strain #009071; B6;129-Ppiftm1Jmol/J). The study mice were bred true from homozygous WT and KO breeders, respectively, which had been generated from heterozygous breeders. All studies were performed exclusively in male mice, which were group-housed with a 12 h light, 12 h dark cycle (7am-7pm) at 22 °C and maintained on a standard chow diet (Harlan Teklad LM-485) or high-fat diet (Research Diets D12492 (60% fat, New Brunswick, NJ, USA), with ad libitum access to food and water. High-fat diet feeding was initiated at 20.9 ± 0.3 weeks of age and continued for 34 weeks.

Fat and lean mass were quantified using NMR technology (EchoMRI, Houston, TX, USA). Energy expenditure, respiratory quotient, food intake, and locomotor activity were assessed using a combined indirect calorimetry system (TSE Systems GmbH, Bad Homburg, Germany) as described previously [8]. Mice had ad libitum access to food and water and were acclimated to metabolic cages for at least 24 h followed by the simultaneous measurement over a 72‑hour period. Intraperitoneal glucose and insulin tolerance testing (GTT/ITT) was conducted in mice subjected to 6 h of fasting and injected intraperitoneally with 1.5 g glucose / kg body weight (20% D-glucose (Sigma) in 0.9% saline) for the GTT, and 1.0 U insulin / kg body weight (Humolog, Lily, Indianapolis, USA) for the ITT. All studies were approved by and performed according to the guidelines of the Institutional Animal Care and Use Committee of the University of Cincinnati, USA.

### Mitochondrial isolation and Seahorse^™^ respirometry

Mitochondria were isolated from quadriceps muscle, brown adipose tissue (BAT), and liver. Tissue samples were excised and transferred into 2‑ml microcentrifuge tubes containing 1 ml of Isolation Buffer 1 (IB1: 210 mM mannitol, 70 mM sucrose, 5 mM HEPES, 1 mM EGTA, 0.5% fatty‑acid–free BSA). Samples were homogenized and centrifuged at 700 × g for 10 min. The supernatant was collected and centrifuged at 14,000 × g for 10 min, and the resulting pellet was resuspended in fresh IB1 and centrifuged again at 10,000 × g for 10 min. This wash step was repeated using Isolation Buffer 2 (IB2: 210 mM mannitol, 70 mM sucrose, 10 mM MgCl₂, 5 mM potassium diphosphate, 10 mM MOPS, 1 mM EGTA). The final mitochondrial pellet was resuspended in 50–200 µl IB2.

Mitochondrial oxygen consumption rate (OCR) was measured using an XF‑24 extracellular flux analyzer (Seahorse Bioscience) following a 10‑min calibration at 37 °C. Isolated mitochondria (15 µg per well) were loaded into XF‑24 plates in 500 µl MAS‑1 buffer (70 mM sucrose, 220 mM mannitol, 5 mM KH₂PO₄, 5 mM MgCl₂, 50 mM KCl, 2 mM HEPES, 1 mM EDTA, 0.1% fatty‑acid–free BSA, pH 7.4). Sequential injections of substrates and modulators were used to define respiratory states: State II (5 mM glutamate, 5 mM malate), State III (0.25 mM ADP), State IV₀ (1 µM oligomycin), and maximal uncoupled respiration induced by FCCP (300 nM). OCR traces were used to calculate basal respiration, ADP‑stimulated State III respiration, oligomycin‑inhibited State IV₀ respiration, and FCCP‑stimulated maximal respiration. Respiratory control ratios (RCRs) were determined as State III/State IV₀ OCR to assess mitochondrial coupling efficiency across genotypes and dietary conditions.

Citrate synthase activity in mitochondrial isolates from quadriceps, BAT, and liver was quantified spectrophotometrically according to the manufacturer’s instructions (CS0720 kit, Sigma-Aldrich, St. Louis, MO, USA).

### Statistical analysis

Statistical analyses were performed using GraphPad Prism (V.9 or higher) or SPSS (V31). Longitudinal comparisons of body weight and percentage weight gain, and glucose excursions in the GTT and ITT, were performed using Two-Way RM ANOVA followed by Sidak’s multiple comparisons test. AUC values and differences in weight, fat and lean mass, fat and lean mass gain, food intake, respiratory quotient and locomotor activity between genotypes within each dietary condition or light/dark phase were evaluated using two-tailed unpaired t-tests. Energy expenditure values were assessed by Analysis of Co-Variance (ANCOVA), using body weight or lean mass as covariates. Results are shown as means ± SEM.

## Results

### Higher weight and fat mass in adult *Ppif* KO mice on SD

**Fig. 1:**
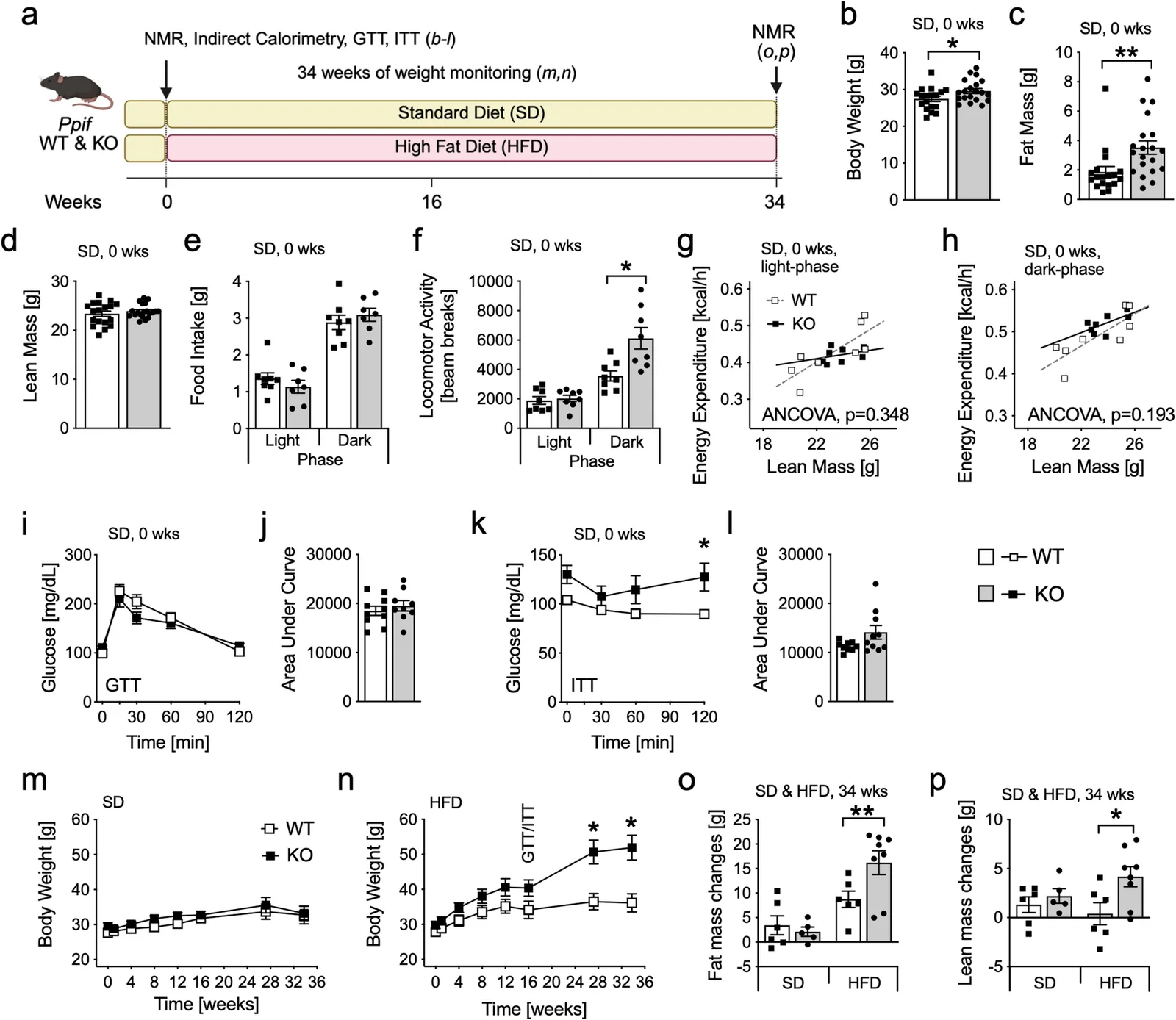
Experimental paradigm, metabolic phenotyping of standard diet-fed *Ppif* WT and KO mice, and longitudinal body weight and body composition trajectories during high-fat-diet feeding.** a**) Experimental paradigm for the metabolic characterization of SD- or HFD-fed male *Ppif* WT and KO mice for a total of 34 weeks, with arrows indicating the times when nuclear magnetic resonance (NMR) measurements, combined indirect calorimetry and glucose tolerance or insulin tolerance tests (GTT/ITT) were performed. Body weight (**b**), fat mass (**c**) and lean mass (**d**) of SD-fed *Ppif* WT and KO mice at week 0 (*n* = 18–20, age 20.9 ± 0.3 weeks). Subgroups of SD-fed WT and KO mice (*n* = 8) were subjected to combined indirect calorimetry (*n* = 8) to assess food intake (**e**), locomotor activity (**f**) or energy expenditure, statistically assessed by ANCOVA and displayed as linear regression plot of light-phase (**g**) and dark-phase (**h**) energy expenditure vs. covariate lean mass. Glucose excursions (left panel) and area under the curve values (AUC, right panel after glucose (**I**,** j**) and insulin (**k**,** l**) tolerance tests (GTT/ITT, *n* = 8–10). Body weight trajectories (**m**,** n**) and gain of fat (**e**) and lean mass (**f**) in *Ppif* WT and KO mice (*n* = 5–10) exposed to SD or HFD for 34 weeks. Two-tailed t-tests (**b**-**f**, **j**, **l**, **o**, **p**), ANCOVA with covariate lean mass (**g**, **h**) and 2-Way RM ANOVA with Šídák’s multiple comparisons tests (**I**, **k**, **m**, **n**). Means ± SEM. *p < 0.05, **p < 0.01

To investigate the impact of *Ppif*-deficiency on the development of adult-onset obesity, we conducted body composition analysis, indirect calorimetry, and glucose homeostasis testing in 20.9-weeks-old *Ppif* WT and KO mice fed standard diet (SD; week 0; Fig. 1a). KO mice exhibited significantly higher body weight (Fig. 1b) and fat mass (Fig. 1c), while lean mass was comparable between genotypes (Fig. 1d). Although food intake remained unchanged, locomotor activity during the dark phase was significantly increased in KO mice relative to WT mice (Fig. 1e, f). Energy expenditure, adjusted for lean mass and assessed by analysis of covariance (ANCOVA), was comparable between genotypes during the light (F(1,13) = 0.948, *p* = 0.348) and dark (F(1,13) = 1.884, *p* = 0.193) phases (Fig. 1g, h). Glucose tolerance, evaluated using a glucose tolerance test (GTT), showed no difference between genotypes, with comparable glucose excursions and area under the curve (AUC) values (Fig. 1i, j). However, during the insulin tolerance test (ITT), blood glucose levels at the 120-minute timepoint were significantly higher in KO mice compared to WT mice (Fig. 1k), although the overall AUC values did not reach statistical significance (Fig. 1l). Together, our data show that *Ppif* KO mice on SD display modestly increased body weight and fat mass, elevated dark-phase locomotor activity, and a slight impairment in late-phase insulin tolerance, without noticeable differences in food intake, energy expenditure, or overall glucose tolerance.

### HFD exposure escalates adult-onset obesity in *Ppif*-deficient mice

We next divided *Ppif* WT and KO mice into two weight-matched cohorts, each receiving either SD or HFD, and monitored body weight and body composition over a period of 34 weeks. Under SD, body weight trajectories and percentage weight gain were comparable between WT and KO mice throughout the 34-week-monitoring (Fig. 1m). Under HFD, KO mice showed significantly greater body weight from weeks 28 to 34 (Fig. 1n). After 34 weeks, fat mass gain was greater in HFD-fed KO compared to WT mice (Fig. 1o), while no significant difference in fat mass gain was observed between genotypes under SD. Lean mass gain was also significantly greater in HFD-fed KO relative to WT mice (Fig. 1p), with no significant difference between genotypes under SD. Collectively, these results indicate that *Ppif* deficiency worsens diet-induced obesity, leading to a greater accumulation of body weight, fat mass, and lean mass specifically in response to HFD exposure.

### Indirect calorimetry does not reveal differences in metabolic homeostasis in SD-fed *Ppif*-deficient mice

**Fig. 2:**
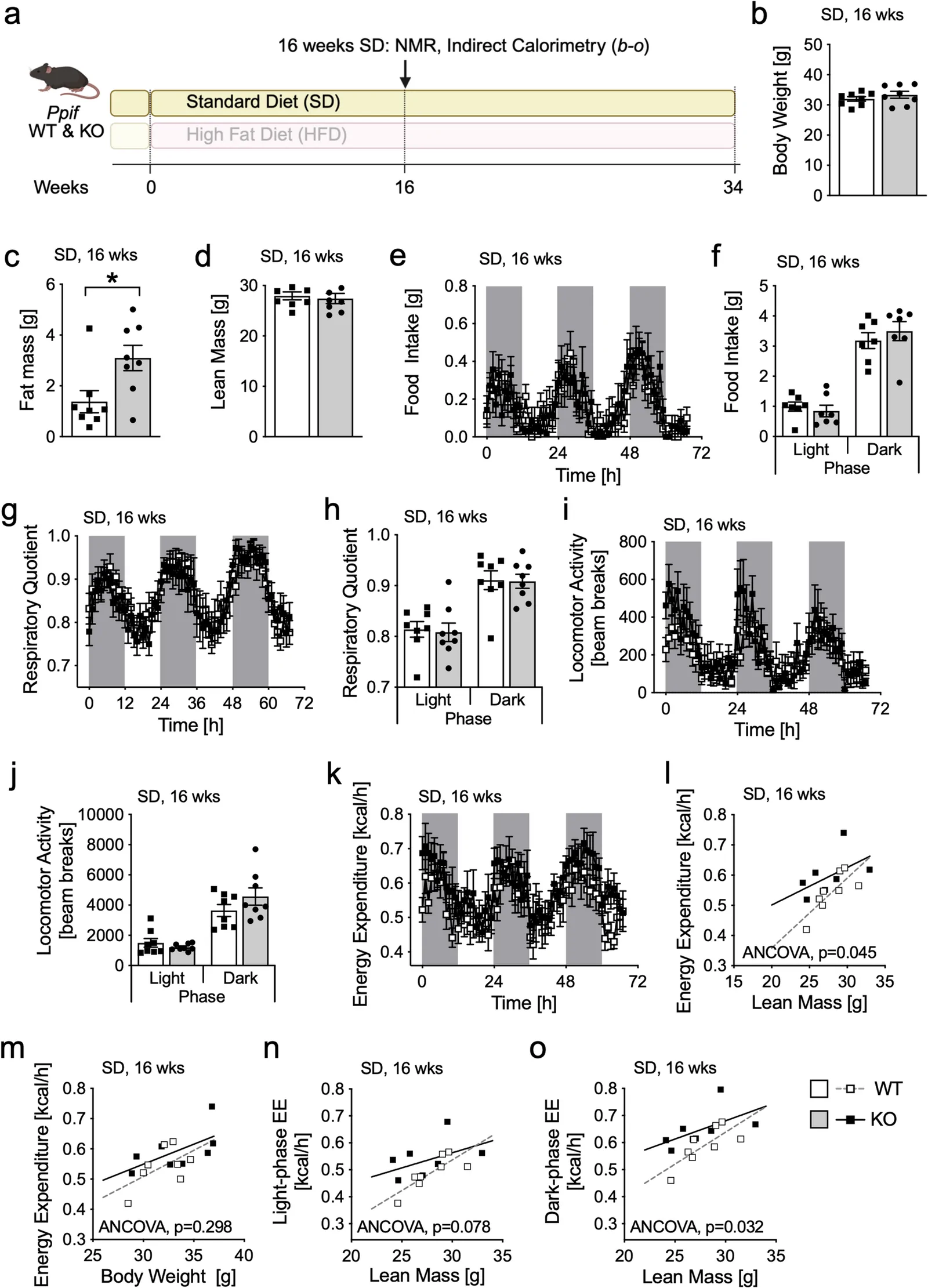
Deep metabolic phenotyping of *Ppif* WT and KO mice after 16 weeks of SD.** a**) Experimental paradigm for the nuclear magnetic resonance (NMR) measurements and combined indirect calorimetry of male *Ppif* WT and KO mice after 16 weeks of SD feeding. Body weight (**b**), fat mass (**c**) and lean mass (**d**) of *Ppif* WT and KO mice (*n* = 8). Subsequent combined indirect calorimetry monitoring over 67 h, averaged for light- and dark-phases, revealed unchanged food intake (**e**,** f**), respiratory quotients (**g**,** h) **and locomotor activity (**i**,** j**). Energy expenditure (EE) traces (**k**) were subjected to ANCOVA and plotted as average EE vs. lean mass (**l**) or body weight (**m**), or light- (**n**) and dark-phase (**o**) EE vs. lean mass. Two-tailed t-tests (**b**-**d**, **f**, **h**, **j**). ANCOVA with covariates lean mass (**l**, **n, o**) or body weight (**m**). Means ± SEM. *p < 0.05

To determine whether the initially observed increase in adiposity in *Ppif*-deficient mice on SD at week 0 was accompanied by alterations in energy homeostasis, we repeated indirect calorimetry monitoring after 16 weeks of SD feeding (Fig. 2a). Body weights were comparable between genotypes (Fig. 2b), whereas fat mass was moderately increased in KO compared to WT mice (Fig. 2c; *p* < 0.05). Lean mass remained comparable between groups (Fig. 2d). Food intake patterns were similar between WT and KO mice over 72 continuous hours (Fig. 2e). Likewise, phase-averaged food intake during the light and dark phases did not differ significantly between genotypes (Fig. 2f). Respiratory quotient (RQ) monitoring displayed the expected diurnal oscillation (Fig. 2g) and comparable phase-averaged RQ values during the light and dark phase between WT and KO mice (Fig. 2h). Similarly, locomotor activity followed a diurnal pattern in both genotypes (Fig. 2i), and both light and dark phase locomotion were comparable between groups (Fig. 2j). Energy expenditure (EE) traces over 72 h demonstrated the expected higher activity during the dark phase in both groups (Fig. 2k). ANCOVA revealed that lean mass was a significant predictor of the average EE (F(1,13) = 8.880, *p* = 0.011), and genotype also exerted a significant main effect (F(1,13) = 4.927, *p* = 0.045), visualized by the regression plot of EE vs. lean mass (Fig. 2l). When adjusted for body weight (F(1,13) = 7.165, *p* = 0.022), the significant genotype effect was no longer present (Fig. 2m; F(1,13) = 1.177, *p* = 0.298). Phase-averaged EE, after adjustment for lean mass, showed a trend toward a genotype effect during the light phase (F(1,13) = 3.661, *p* = 0.078), and was significantly higher in the dark phase (F(1,13) = 5.733, *p* = 0.032) in *Ppif* KO mice (Fig. 2n, o). In summary, these findings indicate that *Ppif* KO mice on standard diet at 16 weeks show a modest yet significant increase in fat mass and energy expenditure, but no detectable differences in body weight and lean mass, or food intake, substrate utilization and locomotor activity, compared to WT controls.

### *Ppif*-deficient mice are more prone to develop high fat diet-induced obesity

**Fig. 3:**
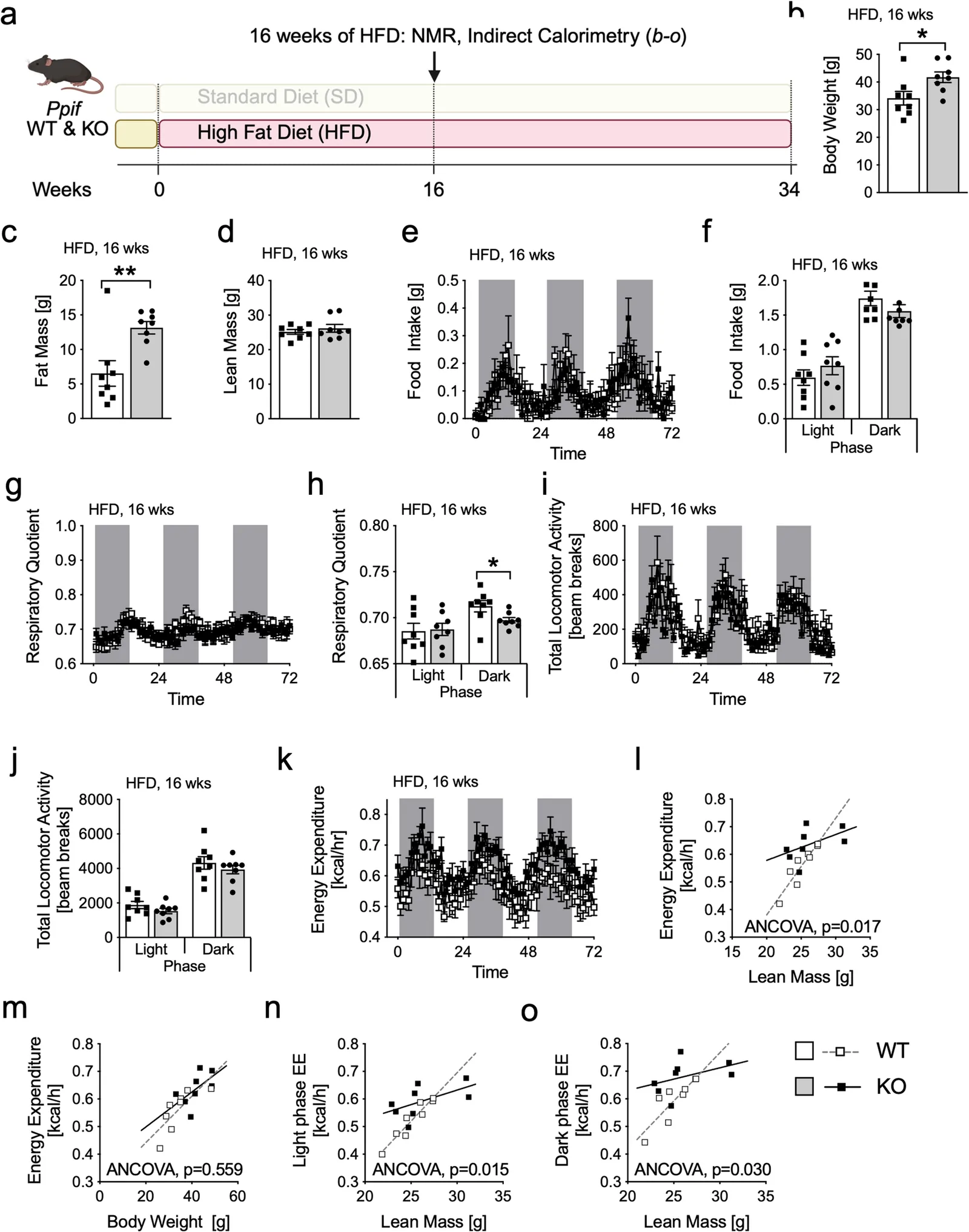
Deep metabolic phenotyping of *Ppif* WT and KO mice after 16 weeks of HFD.** a**) Experimental paradigm for the nuclear magnetic resonance (NMR) measurements and combined indirect calorimetry of male *Ppif* WT and KO mice after 16 weeks of HFD feeding. Body weight (**b**), fat mass (**c**), and lean mass (**d**) of *Ppif* WT and KO mice (*n* = 8). Subsequent indirect calorimetry over 72 h, averaged for light‑ and dark‑phases, revealed unchanged food intake (**e**,** f**), a significant reduction of dark‑phase respiratory quotient in KO mice (**g**,** h**), and comparable locomotor activity (**i**,** j**). Energy expenditure (EE) traces (**k**) were analyzed by ANCOVA and plotted as average EE versus lean mass (**l**) or body weight (**m**), as well as lean‑mass–adjusted EE during light (**n**) and dark (**o**) phases. Two‑tailed t‑tests were used for **b**-**d**, **f**, **h**, **j**; ANCOVA with covariates lean mass (**l**, **n**, **o**) or body weight (**m**). Means ± SEM. *p < 0.05, **p < 0.01

After 16 weeks of HFD feeding (Fig. 3a), *Ppif* WT and KO mice showed significantly increased body weight and fat mass compared with WT mice (Fig. 3b, c). Lean mass was comparable across genotypes (Fig. 3d). Continuous 72-hour food intake monitoring (Fig. 3e) revealed comparable average food intake during the light and dark phases (Fig. 3f). Respiratory quotient traces over 72 h showed comparable oscillations between WT and KO mice (Fig. 3g); however, the phase-averaged RQ during the dark phase was significantly lower in KO mice (Fig. 3h), indicating a higher utilization of lipids as fuel substrate. Total locomotor activity over 72 h showed comparable patterns between groups (Fig. 3i), and phase-averaged locomotor activity during both light and dark phases was not different between genotypes (Fig. 3j). However, total energy expenditure (EE) over 72 h (Fig. 3k) appeared higher in knockout (KO) mice compared to wild-type (WT) mice. ANCOVA, after adjusting for lean mass, confirmed a significant effect of genotype (F(1, df) = 7.709, *p* = 0.017), with lean mass being a strong predictor of EE (F(1, df) = 19.597, *p* < 0.001). Furthermore, a significant Genotype × Lean Mass interaction (F(1, df) = 6.547, *p* = 0.025) indicated differing EE scaling between genotypes, which was visualized by differing slopes for the linear regression of total EE versus lean mass (Fig. 3l). Of note, adjustment for body weight in the ANCOVA did not yield significance between genotypes, indicated by largely overlapping regression lines for EE plotted vs. body weight (genotype: F(1,12) = 0.280, *p* = 0.606; body weight: F(1,12) = 12.210; *p* = 0.004, genotype × body weight: F(1,12) = 0.237, *p* = 0.635, demonstrated also by largely overlapping regression lines for EE plotted vs. body weight (Fig. 3m). Lean mass-adjusted ANCOVA analyses and regression plots (Fig. 3n, o) further revealed higher light phase EE (genotype: F(1,12) = 8.020, *p* = 0.015; lean mass: F(1,12) = 23.906, *p* < 0.001) and higher dark-phase EE (genotype: F(1,12) = 6.018, *p* = 0.030; lean mass: F(1,12) = 13.041, *p* = 0.004) with a significant interaction between genotype and lean mass (light phase EE: F(1,12) = 6.944, *p* = 0.022; dark-phase EE: F(1,12) = 5.020, *p* = 0.045). Taken together, these data reveal that HFD-fed *Ppif* KO mice develop greater obesity than WT mice and exhibit selectively increased dark-phase respiratory quotient and light- and dark-phase energy expenditure without differences in food intake or locomotor activity.

### Comparable glucose homeostasis in *Ppif* WT and KO mice on SD and HFD

**Fig. 4:**
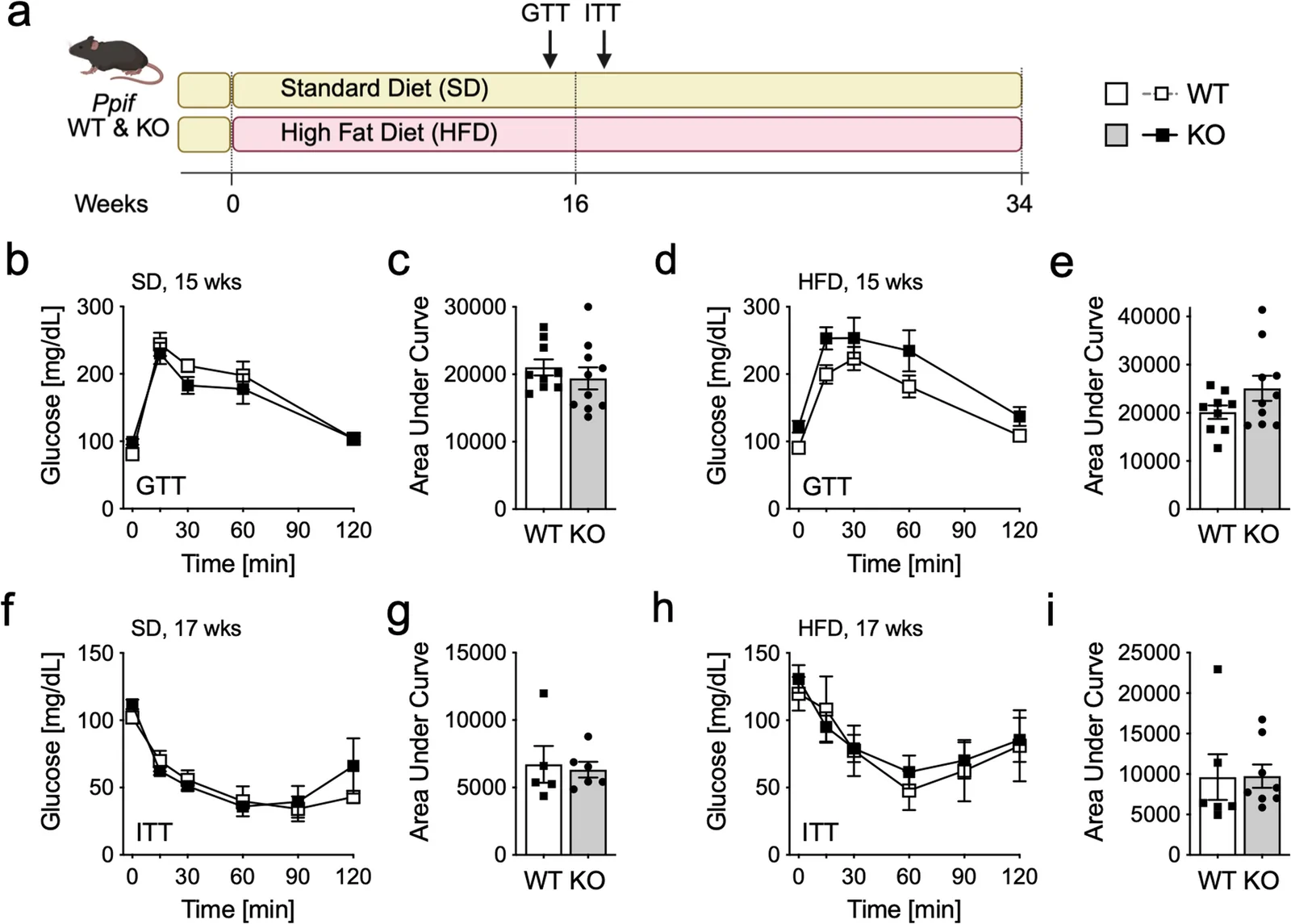
Glucose tolerance and insulin sensitivity in Ppif WT and KO mice fed SD and HFD.** a**) Experimental paradigm: SD and HFD-fed WT and KO mice were subjected to glucose tolerance (**b-e**, 15 weeks of diet exposure, *n* = 9–10) or insulin tolerance tests (**f-i**, 17 weeks of diet exposure, *n* = 5–8). Area under curves values were statistically assessed using two-tailed t-tests (**c**, **e**, **g**, **i**), glucose excursions by 2-Way RM ANOVA with Šídák’s multiple comparisons tests (**b**, **d**, **f**, **h**). Means ± SEM

At week 15 of SD exposure (Fig. 4a), intraperitoneal GTTs revealed comparable glucose excursions and AUC values in WT and KO mice (Fig. 4b, c). In HFD-fed mice, both groups had more pronounced and higher glucose excursions compared to those on the SD. Although the AUC for the KO group seemed higher than that of the WT group, there were no statistically significant differences detected between the two genotypes (Fig. 4d, e; Two-Way Repeated Measures ANOVA left panel: *p* = 0.088; t-test right panel: *p* = 0.12). ITTs at week 17 of diet exposure in SD-fed mice revealed a comparable decrease in blood glucose following insulin injection, with no significant differences in AUC values between groups (Fig. 4f, g). In the HFD-fed ITT, both groups exhibited a blunted glucose-lowering response relative to SD-fed animals, yet AUC values again were not different between WT and KO mice (Fig. 4h, i). Collectively, these results indicate that *Ppif* deficiency did not significantly alter glucose tolerance or insulin sensitivity under either SD or HFD conditions at these timepoints.

### Elevated state 3 respiration in quadriceps mitochondria from *Ppif* KO mice

**Fig. 5:**
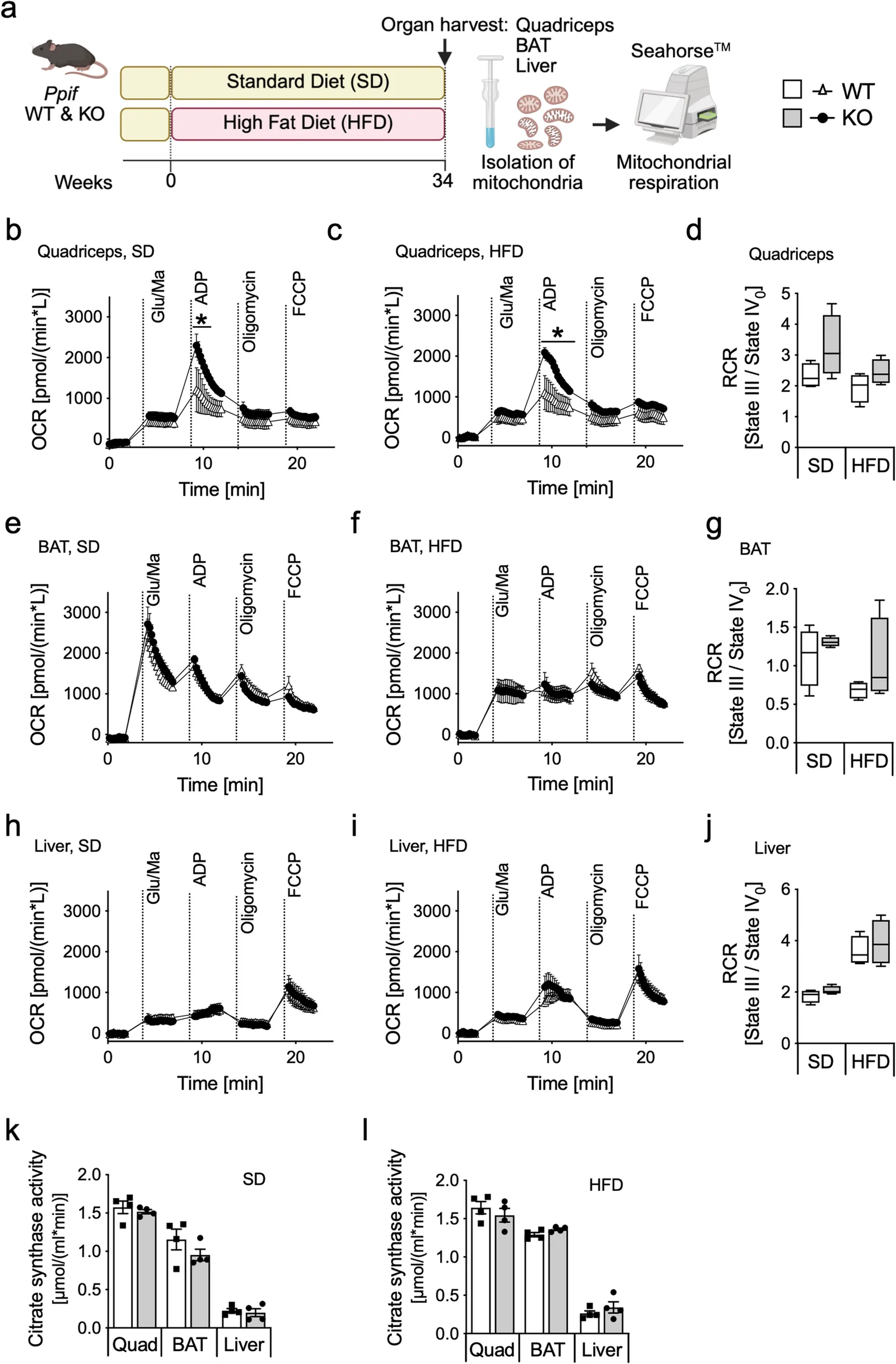
Mitochondrial respiration in skeletal muscle, brown fat and liver in *Ppif* WT and KO mice fed SD or HFD. **a**) Experimental paradigm for the organ harvest of male *Ppif* WT and KO mice exposed to SD or HFD for 34 weeks, followed by the isolation of mitochondria and measurement of oxygen consumption rates (OCR; **b**,** c**, **e**,** f**, **h**,** I**), respiratory control ratios (RCR; **d**,** g**, **j**) and citrate synthase activities (**k**,** l**) in in the quadriceps (**b-d**), BAT (**e-g**) and livers (**h-j**). Two-tailed t-tests (**d**, **g**, **j**, **k**, **l**) and multiple t-tests with Holm-Sidak correction for multiple comparisons (**b, c, e, f, h, i**). Mice used for mitochondrial isolation & measurement: *n* = 4 per genotype. Means ± SEM. *p < 0.05

To determine if the metabolic phenotype of *Ppif* KO mice was associated with differences in mitochondrial respiratory function or mitochondrial content, we conducted mitochondrial respiration assays and measured citrate synthase activity in isolated mitochondria from the quadriceps, brown adipose tissue (BAT), and liver of WT and KO mice fed SD or HFD for 34 weeks (using four randomly selected mice per group; Fig. 5a). At the time of sacrifice, the surviving *Ppif* WT (*n* = 6) and KO mice (*n* = 5) on SD had comparable weight, fat and lean mass, respectively. *Ppif* KO mice on HFD (*n* = 8) had higher weights (Fig. 1n), fat mass (KO: 20.74 ± 2.31, WT: 10.45 ± 1.77; *p* = 0.0041) and lean mass (WT: 22.83 ± 1.08 g, KO: 27.95 ± 1.36 g; *p* = 0.012) compared to their WT counterparts (*n* = 6). In quadriceps mitochondria, ADP-stimulated State III respiration was significantly increased in KO mice compared to WT mice under both SD (Fig. 5b) and HFD (Fig. 5c), while no additional significant differences were detected at other respiratory states. Respiratory control ratio (RCR) values in quadriceps were not different between genotypes under either dietary condition (Fig. 5d). In BAT, oxygen consumption rate (OCR) profiles were comparable between WT and KO mice under SD (Fig. 5e) or HFD (Fig. 5f), and RCR values were similarly comparable between genotypes under both diets (Fig. 5g). In liver, OCR profiles under both SD and HFD and RCR values under both dietary conditions were comparable between WT and KO mice (Fig. 5h–j). Citrate synthase activity was not significantly different between WT and KO mice across all three tissues under SD (Fig. 5k) or HFD (Fig. 5l) conditions, indicating comparable mitochondrial content between genotypes. Across all tissues and dietary conditions, liver mitochondria showed the lowest citrate synthase activity, while quadriceps and BAT exhibited higher and relatively similar levels. In summary, these findings indicate that *Ppif* deficiency selectively increased ADP-stimulated mitochondrial respiration in quadriceps under both dietary conditions, without broadly altering mitochondrial coupling efficiency or mitochondrial content across metabolically active tissues.

## Discussion

This study shows that whole‑body *Ppif* deficiency exacerbates age-onset obesity when 20-week-old mice are exposed to HFD for up to 34 weeks. Under SD, *Ppif*‑deficient mice displayed modestly elevated fat mass early in adulthood, despite unchanged food intake, lean mass, and weight trajectories. Importantly, EE, adjusted for lean mass and analyzed by ANCOVA, was higher in *Ppif*‑deficient mice both under SD and HFD, with a significant genotype × lean mass interaction in HFD-fed KO mice, indicating that lean‑mass‑derived tissues, especially skeletal muscle, disproportionately contribute to EE differences.

Direct mitochondrial phenotyping supports this interpretation. After 34 weeks of HFD, obese *Ppif*‑deficient mice showed selectively increased ADP‑stimulated oxidative phosphorylation in quadriceps, but not in BAT or liver, without changes in mitochondrial abundance or coupling efficiency. These data indicate that CypD loss enhances skeletal muscle oxidative responsiveness, while other metabolic tissues remain comparatively unaffected. Together, these findings establish that CypD shapes whole‑body metabolism through diet‑dependent and tissue‑specific effects, particularly within skeletal muscle.

### CypD, mPTP sensitization, and tissue‑specific mitochondrial respiration

CypD regulates the Ca²⁺ and redox sensitivity of the mitochondrial permeability transition pore (mPTP), lowering the threshold for pore opening and thereby influencing cell survival, ROS signaling, and metabolic flexibility [3–5]. Consequences of CypD deletion are strongly context‑dependent. For instance, CypD is essential for necrotic cell death in Ca²⁺‑overloaded dystrophic muscle [9], but dispensable for denervation‑induced atrophy [10]. CypD deletion was linked with the protection of mitochondria from metabolic or Ca²⁺ stress [6, 7]. Our findings align with this model of conditional mPTP involvement. ADP‑stimulated respiration in the quadriceps of HFD-fed *Ppif*‑deficient mice was elevated, which suggests that reduced mPTP susceptibility may enhance mitochondrial stability during sustained nutrient excess. However, our measurements were performed in isolated mitochondria and should be interpreted with caution. Likewise, due to the need to process multiple tissues immediately after euthanasia, our respiration assays were conducted using a common protocol optimized for liver mitochondria. Consequently, FCCP concentrations were likely suboptimal for skeletal muscle and BAT mitochondria, limiting interpretation of maximal uncoupled respiration. Similarly, BAT mitochondria were assayed without GDP-mediated inhibition of UCP1, precluding rigorous assessment of coupled respiration and respiratory control ratios in this tissue. Accordingly, future studies employing tissue-optimized protocols and complementary approaches such as permeabilized muscle fibers will be required to validate and extend these findings.

### CypD and mitochondrial fuel selection

CypD loss‑ and gain‑of‑function have been linked to altered metabolic substrate preference. Proteomic profiling of CypD‑null hearts revealed broad remodeling of the TCA cycle, branched‑chain amino acid catabolism, β‑oxidation, and pyruvate metabolism [11]. Other studies report that CypD deficiency biases toward greater reliance on carbohydrate metabolism and reduced fatty‑acid oxidation [12, 13]. In pancreatic β‑cells, reduced mPTP opening enhances coupling efficiency and insulin secretion [14], further implicating CypD in the regulation of metabolic substrates.

Our long‑term phenotyping aligns with this metabolic shift. *Ppif*‑deficient mice accumulated more fat mass on both SD and HFD without consuming more calories, indicating altered metabolic partitioning rather than hyperphagia. Together with the observed increase in skeletal muscle oxidative phosphorylation, without accompanying changes in mitochondrial content, this pattern may suggest a preferential use of glucose‑supported respiration. Whether such a metabolic role of CypD is consistent with the AMPK‑driven remodeling described in other CypD‑null models [12], remains to be determined.

### Tissue‑specificity of CypD effects

CypD action varies markedly across tissues. The heart, which expresses high CypD levels, shows strong CypD‑dependent regulation of mPTP activity, Ca²⁺ handling, and susceptibility to heart failure [15]. In contrast, skeletal muscle exhibits relatively low CypD dependence at rest but stronger involvement during activity‑induced Ca²⁺ flux [16]. Liver‑specific *Ppif* deletion increases visceral adiposity and glucose intolerance under HFD, whereas muscle‑specific deletion has minimal effects [17].

Our metabolic phenotyping and respirometry data mirror this tissue‑selective pattern. Only the quadriceps showed increased ADP-stimulated State III respiration in *Ppif*‑deficient mice, the BAT and liver did not. This skeletal‑muscle‑restricted enhancement of oxidative phosphorylation may explain why EE, adjusted for lean mass, increases in *Ppif*‑deficient mice, yet adiposity still rises, as non‑muscle tissues likely exhibit altered thermogenic, survival or storage properties upon *Ppif* deletion than muscle.

CypD also has direct roles in adipocyte biology. High‑fat diet induces adipocyte cell death through a CypD‑dependent intrinsic pathway [18], and CypD loss protects adipocytes from lipotoxic necrosis [19]. This protection may favor adipose expansion. Together with our findings, this suggests that increased fat mass in *Ppif*‑deficient mice, also under SD feeding, may reflect enhanced adipocyte survival, reduced inflammatory turnover, and greater lipid storage capacity. However, this hypothesis requires further experimental validation.

### Energy expenditure changes upon *Ppif* deletion

A major discrepancy with earlier reports of reduced obesity susceptibility in *Ppif*-deficient mice arises from differences in energy expenditure analysis. Devalaraja‑Narashimha et al. – like us - [19] report elevated energy expenditure, but they normalized VO₂ to total body weight, a ratio‑based approach now recognized as statistically invalid and prone to misleading interpretations. By contrast, applying ANCOVA with lean mass as the covariate, as recommended by Tschöp et al. [20], we reveal that lean mass is the primary determinant of EE and that genotype modifies the EE–lean‑mass relationship in our *Ppif*-deficient mice. However, this significant lean mass x genotype interaction does not necessarily translate into higher energy expenditure in KO versus WT mice when lean mass is comparable, as was the case at both indirect calorimetry time points. This may partly explain the apparent discrepancy between the calorimetry and body weight data, which at first glance appear difficult to reconcile. However, our calorimetry data - together with the increased lean mass observed in HFD-fed KO mice – identifies skeletal muscle as a key tissue for future investigation and raises the intriguing possibility that CypD may play a previously unrecognized role in age-related skeletal muscle remodeling and potentially sarcopenia.

A limitation in our data is nonetheless the lack of causality between enhanced mitochondrial respiratory capacity, altered energy expenditure and higher adiposity. Future studies are required to clarifying the contribution of these mechanisms, potentially including direct assessments of substrate utilization and metabolic flux, and indirect calorimetry measurements at earlier stages of HFD exposure. Such indirect calorimetry studies, which are subject to technologically inherent limitations in detecting subtle energy expenditure differences between genotypes, primarily quantify oxygen-dependent oxidative metabolism and may thus not capture additional processes contributing to energy balance, such as substrate utilization, metabolic fluxes, futile cycling, or other potential energy-dissipating pathways [21–24]. Accordingly, a potential contribution of such processes to the observed phenotype remains an interesting but untested possibility.

### Discrepant reports on obesity susceptibility in *Ppif* KO mice

Previous studies on the metabolism of whole-body CypD knockout mice have produced inconsistent results. *Ppif*‑deficient mice either displayed protection from diet‑induced obesity [19] or a higher propensity for adult‑onset obesity developing under normal SD conditions [25]. The latter phenotype was further corroborated in liver‑specific *Ppif* KO mice, which developed increased adiposity and glucose intolerance on HFD, whereas skeletal‑muscle–specific deletion produced no differences in body weight or fat mass [17]. Our results align more closely with the studies reporting heightened susceptibility to obesity upon loss of CypD. Differences in genetic background, gut microbiome composition, and housing environment remain unavoidable sources of variation across CypD‑knockout studies, including our own, and may interact with genotype in ways that are difficult to fully control. Such cohort‑specific influences likely contribute to the discrepant metabolic fates reported for global or tissue-specific *Ppif*‑deficient mice.

A clearer understanding of this susceptibility may require deeper analysis of *Ppif* functions in metabolically active tissues. Such studies should entail adipose‑tissue biology in *Ppif*‑deficient mice, including adipocyte size distribution, lipid‑turnover kinetics, macrophage infiltration, and crown‑like structure formation, to determine whether the phenotype reflects cell‑autonomous adipocyte effects, altered adipose immune dynamics [18], or both. Given the divergent phenotypes of liver‑ and muscle‑specific knockouts, adipocyte‑specific and myeloid‑specific conditional *Ppif* deletions could help resolve the tissue contributions to whole‑body adiposity.

## Conclusion

Together, our findings position CypD as a diet‑ and tissue‑specific regulator of energy homeostasis, shaping mitochondrial fuel selection, oxidative capacity, adipocyte survival, and whole‑body metabolic adaptability. Rather than acting as a uniformly protective or detrimental factor, CypD emerges as a context-dependent modulator whose influence on adiposity and mitochondrial function varies with dietary environment, tissue identity, and the energetic demands of individual cell types, consistent with broader models of CypD-dependent metabolic plasticity. The coexistence of enhanced skeletal muscle respiration and increased adiposity in *Ppif*-deficient mice further underscores the complexity of the metabolic phenotype and suggests that alterations in mitochondrial respiratory capacity do not necessarily translate into predictable whole-body metabolic outcomes. These findings highlight the need for additional studies, including tissue-specific *Ppif* knockout models, to define the contributions of skeletal muscle, adipose tissue, and liver to systemic metabolic regulation and obesity susceptibility. Future studies should also include female mice to assess the extent to which these effects are sex dependent. 

## Data Availability

All data supporting the findings of this study are available within the paper.

## Declarations

### Clinical trial number

Not applicable. This study did not involve a clinical trial and was not registered.
